# Supplementary figures and images for: Model-based identification of conditionally-essential genes from transposon-insertion sequencing data
Source: PLoS Comput Biol. 2022 Mar 7;18(3):e1009273. doi: 10.1371/journal.pcbi.1009273 (PMC8929702; doi:10.1371/journal.pcbi.1009273)

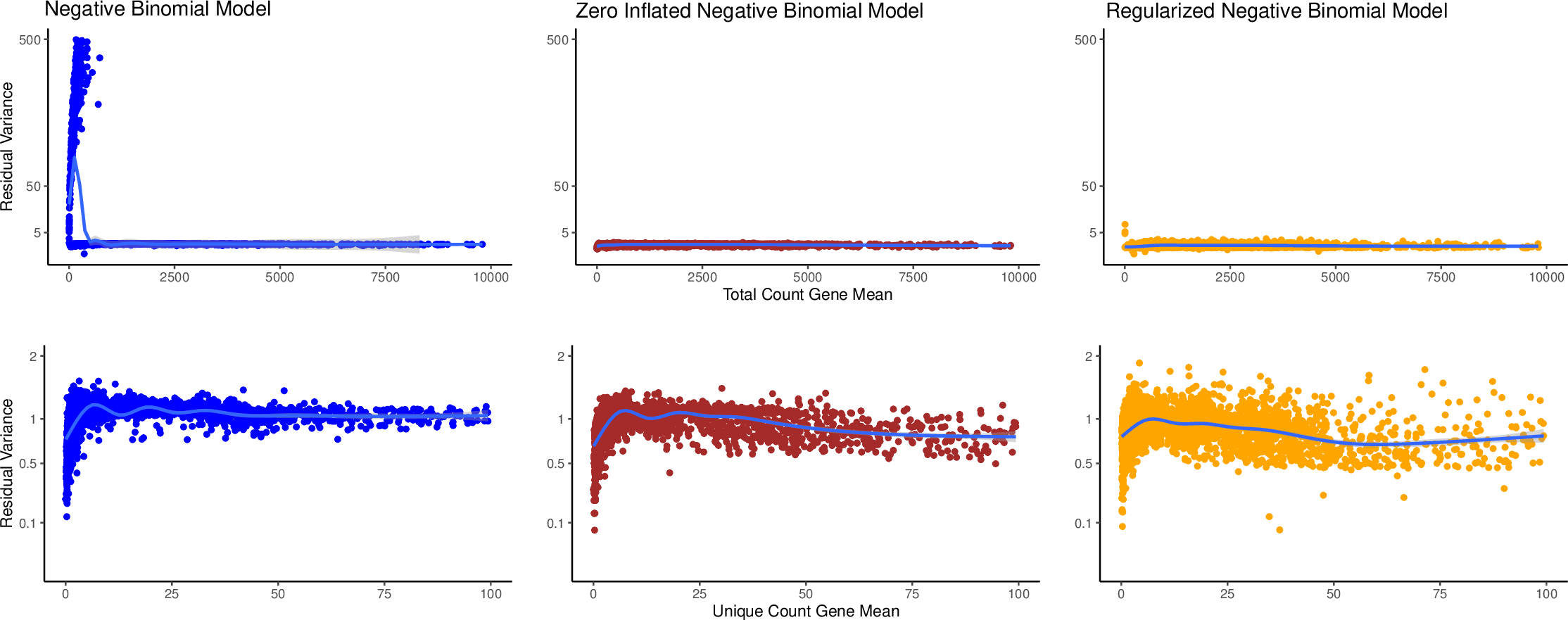

Supplement: S1 Fig — These results show data for only the control and one condition in contrast to the multiple condition results shown in Fig 3. (TIF) [file pcbi.1009273.s004.tif]

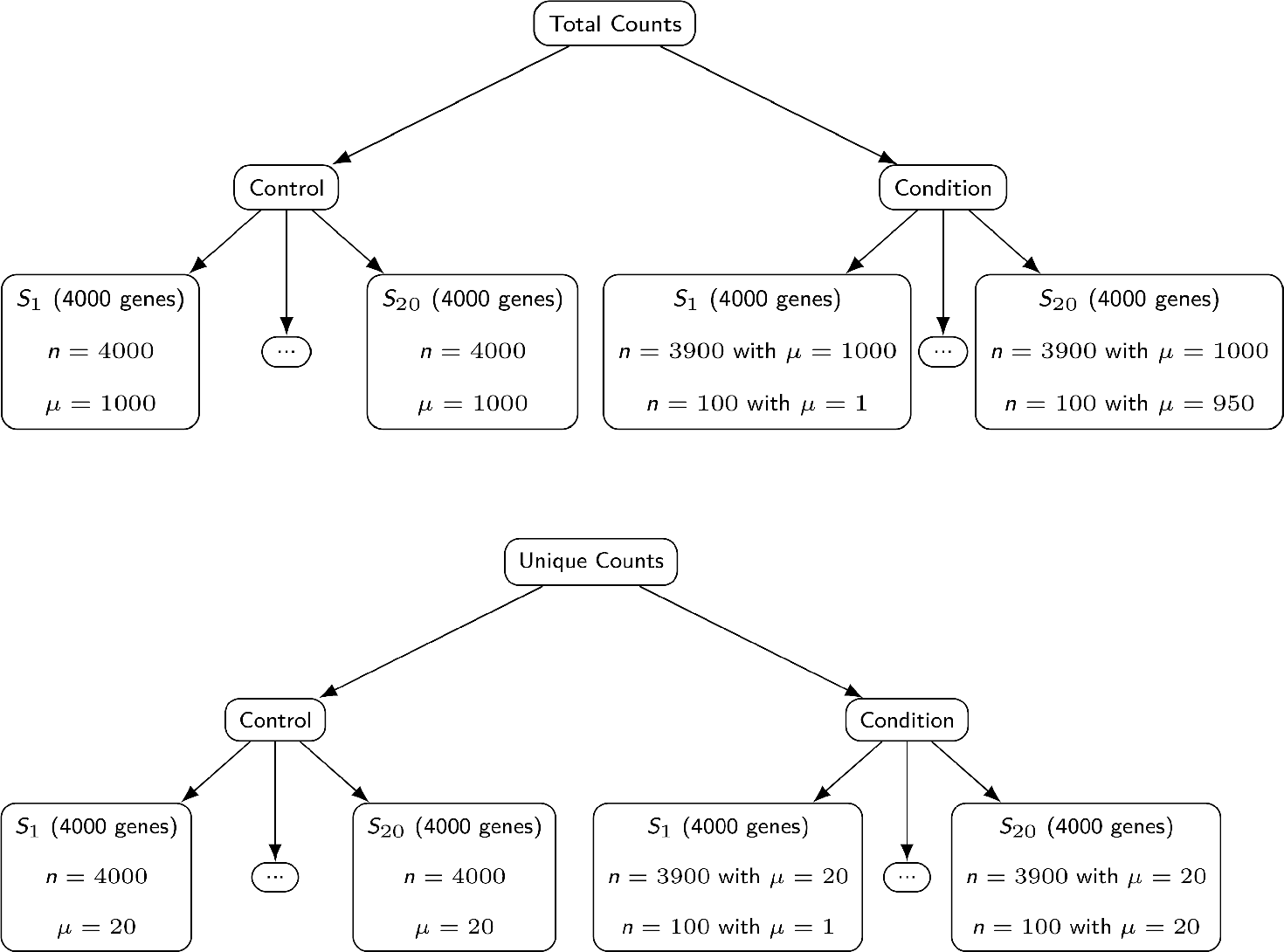

Supplement: S2 Fig — A total of 20 sets each containing 4000 genes in a control condition setup are simulated for total counts and unique counts separately according to schema and parameters shown in the figure. (TIF) [file pcbi.1009273.s005.tif]
