# Supplementary material for: Model-based identification of conditionally-essential genes from transposon-insertion sequencing data
Source: PLoS Comput Biol. 2022 Mar 7;18(3):e1009273. doi: 10.1371/journal.pcbi.1009273 (PMC8929702; doi:10.1371/journal.pcbi.1009273)
Supplement: Attachment — Submitted filename: glbio_reviewer_scomments.pdf [file pcbi.1009273.s006.pdf]

Answers to Reviewer 1

Comment R1.1 *Benchmarking: For a two-condition setup, I would like to see how the method performs compared to Subramaniyam et al.'s ZINB approach as well as the older techniques based on count normalization. It would be comforting to know that the model at least matches the results of previous approaches in this limited context; its contributions to the more-complex multi-condition setup would then be more meaningful.*
Response to R1.1 We have performed a comparison of the two-condition setup of our model using the simulated data. Our model performs comparably well in a two-condition setup. We have included the results from two-condition setup in the Supplementary Figure 1.

Comment R1.2 *Notation: this needs substantial cleanup. In trying to be general, the authors have introduced a surfeit of subscripts and superscripts which are not entirely consistent and hence difficult to track. For example, line 101-103 did not make sense in the context of later analysis in Eqn 1 (line 202)– I remain unclear what the tuple refers to. Similarly, what exactly are x_e and x_g in Eqn 1, and is $x_e x_g$ an element-wise product or a matrix multiplication?*
Response to R1.2 The notation in these sections was unclear and we have clarified it. On lines 101-103, we have eliminated the reference to the locations as the raw data. It was unnecessary to the rest of the paper. We have also eliminated the extraneous reference to the experimental index j as it also was unnecessary for the rest of the paper. On line 202, we have recast the standard linear regression model using more standard notation. We have clarified that the model matrix terms are indeed indicator matrices; these are also called dummy variable matrices in statistics, though we have refrained from using that term. We have carefully reviewed the notation throughout the paper to ensure consistency.

Comment R1.3 *Fig 1: in the context of my previous point, it would be much more helpful if Fig 1 was annotated with numbers that relate back to the notation, indicating what y_{ger} or y^{tot} or y^{uniq} are in this specific example. Right now, the figure feels a bit like a cryptic puzzle that needs to be decoded. In lines 210-213, why say "excess or depletion" and not choose one of those terms? Maybe just put arrows on the figure showing excess for stress conditions (in wt) and depletion δlon?*
Response to R1.3 We have modified our Fig 1 to clearly explain the nested experimental design of the transposon sequencing experiments. At the higher level, each experiment has several genetic backgrounds(g) and environmental conditions(e) which are nested within the genetic background. Each of the nested environmental conditions(e) has several replicates.

.

Comment R1.4 *it seems to me that neither of these β thresholds are symmetric around zero– the threshold of significance for negative beta seems lower than that for positive beta. That's not what Eqn 3 would suggest?*
Response to R1.4 Symmetry around the coefficients is generally not expected because a nested condition may have substantially more essential genes than dispensable genes. An example would be a high-stress condition that may result in suppressing the growth of bacteria by the inactivation of multiple genes. You would expect more essential gene effects than dispensable. Since we are using a local FDR procedure, some z-values are much more extreme than others and not all hypotheses are equally likely to be contributing to the false discoveries. It is not uncommon to see the asymmetry in local false discoveries

Comment R1.5 *Eqn 1: Was the choice to also have* x_e *as a separate term explored? Why not have it? Do the results change a lot?*
Response to R1.5 We did explore including x_e as a separate term, however, the subsequent interpretation of the model coefficients did not accord well with the scientific questions of interest. While the model is still identifiable with the construction suggested, the downstream hypothesis testing step is more complicated because a sum of coefficients must be tested rather than a single coefficient. The scientific questions are more directly addressed by the nested model.

Answers to Reviewer 2

Comment R2.1 *It would be nice to see how well this model performs in comparison to others. Also, if you can you should make your code available on CRAN or Bioconductor.*
Response to R2.1 We have compared our model to two other popularly used tools, Zero-inflated negative binomial model as implemented in [1] is part of TRANSIT package and negative binomial model as implemented in ESSENTIALS package[2]. We currently have an R package called `rnbtn` which integrates our nested regularized negative binomial model with the local false discovery procedure.

Answers to Reviewer 3

Comment R3.1 *The data set includes only experiments conducted with Caulobacter crescentus. I do not think this is an issue, but I would be curious to know if there is a justification to this limitation (i.e. are data from other organisms available? With same volume/quality?)*
Response to R3.1 We selected Caulobacter because it is a model organism for the protein homeostasis system which is a long term focus of our research. This is not an inherent limitation of the methodological approach though and we have selected an additional organism, Pseudomonas, to validate the methodology for this paper. It is clear from the Figure *3*(D-F) that many genes are called conditionally essential in both the carbon and nitrogen shift conditions while only one essential gene is identified in the stress. This indicates that many conditional genes in carbon/nitrogen are due to the shift in the minimal media similar to what we have shown for the *Caulobacter crescentus.*

Comment R3.2 *Figures could be improved. The caption could be more descriptive and annotation more explicit. Eventually, the font size is too small and Fig. 1 is difficult to read*
Response to R3.2 We have made extensive updates to the figures to increase the font size and descriptiveness of the figure captions.

Comment R3.3 *A figure at the beginning illustrating the principles of Tn-seq experiments and variables used in this paper could improve readability to a broader audience. Though, this is merely a suggestion.*
Response to R3.3 We have modified our Fig 1 to clearly explain the nested experimental design of the transposon sequencing experiments. At the higher level, each experiment has several genetic backgrounds(g) and environmental conditions(e) which are nested within the genetic background. Each of the nested environmental conditions(e—g) has several replicates.

Comment R3.4 *Is the code available? An implementation of the methods should be made public*
Response to R3.4 This is a very good suggestion. We have created an R package called `rnbtn` which integrates our nested regularized negative binomial model with the local false discovery procedure. This package is available on bioconductor and is referenced in the manuscript.

Comment R3.5 *The authors compare their methods to a zero-inflated negative binomial model (similar to [15]) and to unregularized negative binomial model from [11]. It was not clear to me why they say of the same type? Are implementations of [11] and [15] available? Were other software available for a comparison?*
Response to R3.5 We have compared our model to two other popularly used tools, Zero-inflated negative binomial model as implemented in [1] is part of TRANSIT package and negative binomial model as implemented in ESSENTIALS package[2]

Comment R3.6 *The results/prediction remains speculative (this is not a critic though). Further investigations of specific examples could be interesting for an oral presentation (or as an addition to the Discussion/Conclusion)*
Response to R3.6 Thanks for this comment. We have performed competitive validation experiments on one of the genes that we identified as conditionally dispensable in a heat stress environment, clpA — a protease. To our knowledge this is a novel finding that is relevant to our understanding of the protein homeostasis system that was indicated by our transposon experiments

and model. We performed biological replicates in triplicate and added a discussion of these results to the Results section.

References

[1] Siddharth Subramaniyam, Michael A. DeJesus, Anisha Zaveri, Clare M. Smith, Richard E. Baker, Sabine Ehrt, Dirk Schnappinger, Christopher M. Sassetti, and Thomas R. Ioerger. Statistical analysis of variability in TnSeq data across conditions using zero-inflated negative binomial regression. *BMC Bioinformatics*, 20(1):603, November 2019.

[2] Aldert Zomer, Peter Burghout, Hester J. Bootsma, Peter W. M. Hermans, and Sacha A. F. T. van Hijum. ESSENTIALS: Software for Rapid Analysis of High Throughput Transposon Insertion Sequencing Data. *PLOS ONE*, 7(8):e43012, August 2012.
