# Supplementary material for: Model-based identification of conditionally-essential genes from transposon-insertion sequencing data
Source: PLoS Comput Biol. 2022 Mar 7;18(3):e1009273. doi: 10.1371/journal.pcbi.1009273 (PMC8929702; doi:10.1371/journal.pcbi.1009273)
Supplement: Attachment — Submitted filename: ploscomp_reviewers_letter.pdf [file pcbi.1009273.s007.pdf]

Answers to Reviewer 1

Comment R1.1 *One comment to make the manuscript more accessible to its target audience: the gene X environment experimental paradigm the authors investigate is generally referred to as a genetic interaction study – referencing this term in the abstract and manuscript text would increase the ability of those conducting these sorts of experiments to find the method. Additionally, it would probably be worth contrasting the authors' proposed method with DeJesus et al 2017 NAR "Statistical analysis of genetic interactions in Tn-Seq data".*
Response to R1.1 We appreciate the feedback to improve our manuscript. Indeed, our experimental paradigm is a genetic interaction study. We have updated the abstract and manuscript text to make this paper more accessible to the readers.

We included a paragraph in the related work section providing the context of DeJesus et. al. [1] work and some limitations. This should help our target audience in differentiating different experimental paradigms and respective statistical approaches used.

Comment R1.2 *A second comment on terminology: the authors use the terms 'conditionally essential' and 'conditionally dispensable' to refer to genes whose deletion have negative and positive effects on fitness, respectively. The term 'essential' strictly refers to genes whose absence completely abrogates growth in pure culture (not just a fitness defect), though this term is frequently abused in the pooled screening literature so perhaps not much point in fighting the tide. On the other hand, 'dispensable' just means non-essential – I think what the authors mean is something more like 'conditionally detrimental', as all genes with no effect on fitness either way under the assay conditions are clearly dispensable, but probably not especially interesting (at least in the studied condition).*
Response to R1.2 We discussed the usage of terminology with community members actively involved in genetic screening research. With their feedback, we have sought to provide a rigorous mathematical foundation for the terminology. We have added several paragraphs to the problem statement section to clearly define our terms and we use those terms throughout the manuscript. These terms correspond to the parameters outlined by the reviewer's comments.

Our additions begin with the following text: "Let the set of genes under investigation be $\mathcal{S}$ with $m = |\mathcal{S}|$. We partition $\mathcal{S}$ into a set of essential genes, $\mathcal{A}$, and the set of nonessential genes, $\overline{\mathcal{A}} = \mathcal{S} \setminus \mathcal{A}$. Essential genes show no growth when disrupted in a wild-type strain in standard media. Then, we partition the set of nonessential genes into a set of conditionally essential genes, $\mathcal{B} \subseteq \overline{\mathcal{A}}$, and a set of conditionally nonessential genes, $\overline{\mathcal{B}} = \overline{\mathcal{A}} \setminus \mathcal{B}$. The conditionally essential set contains genes that when disrupted still yield growth in control conditions, but do not grow when the genetic background or media conditions are varied; this set depends on the growth condition...." We also added a supplementary figure to clarify these set memberships in hierarchical form.

Comment R1.3 *A final point that could use some clarification is the theoretical basis behind using both total counts and unique counts. I could see an argument based on PCR bias or jackpotting – I don't think this needs to be proven, but it would be nice to have some speculation or intuition on why this is a sensible thing to do besides that it reduces the total number of discoveries. It may also be worth discussing the patterns seen in figure 3 in this context – do the authors have any speculation as to what is happening with the blue and red triangles?*
Response to R1.3 We thank the reviewer for the suggestion. We have now added a paragraph called "Total and Unique Insertions" to the introduction section. Total counts are obtained by

summing up the counts across all the insertion sites in a gene. Total counts tend to reflect the growth of bacteria under a particular condition, which is extremely useful for measuring overall fitness effects. While total counts are a useful measure to identify conditionally beneficial/detrimental genes, they may be skewed when there is an insertion hotspot. Unique counts are obtained by counting the number of insertion sites in a gene. Unique counts are less susceptible to insertion hotspot effects. We also included the interpretation of patterns in figure 3 in the results. Briefly, the intersection of blue and red triangles shows genes that are conditionally beneficial/detrimental by measures of total insertions and unique insertions. They capture differential fitness for both gene-wise insertions akin to growth and site-specific variation, giving much more confidence in functionally annotating a particular genetic component.

Comment R1.4 *Minor point: on page 6, lines 206 and 209, I'm not sure what delta YFG means (besides a gene deletion background by context) – it would be worth either clarifying what this acronym means, or using something that more obviously just a placeholder (e.g. delta gene). Similarly, on the same page, the authors abruptly switch to talking about delta lon with no context – this makes sense later in the manuscript but is a bit confusing at that point.*
Response to R1.4 We appreciate the reviewer for pointing this out. YFG is a typical abbreviation in microbiology standing for "Your Favorite Gene". We now have replaced Δlon background which is a mutant background discussed in our paper instead of using YFG.

Answers to Reviewer 2

Comment R2.1 *Lines 280-1: Naturally taking the intersection will reduce the false positives, but it will also increase the false negatives. In fact, while the authors here mention this intersection, this is not used at all later as the counts for total/unique are presented seperately (see 6 below). The issue of false negatives (or alternative, of power or precision/recall tradeoffs) is never discussed.*
Response to R2.1 We thank the reviewer for their comment. We added a simulation experiment showing a comparison of sensitivity, specificity and accuracy of total counts, unique counts and intersection for the simple Negative Binomial model (NB), Regularized Negative Binomial model (RNB), and Zero-inflated negative binomial (ZINB). We studied each model's variation trend of sensitivity, specificity, and accuracy to changes in condition mean for total counts, unique counts, and intersection. Simulation methods and the results were added to the Sensitivity, Specificity, and Accuracy paragraph. Supplementary Figure 2 depicting the results was added to the manuscript. We also added the intersection calls of our conditionally essential genes in the Tn-seq experiment. Through this experiment, we have shown that taking an intersection of total and unique counts reduces the false positives(higher specificity), but it comes at the expense of false negatives (lower sensitivity) especially as the condition mean becomes closer to the control mean. We discuss this trade-off in the manuscript.

Comment R2.2 *The results of the simulation experiment are only indirect validation as they do not directly address the question of whether the model correctly picks out the ground truth set of essential/dispensable genes, which is the task that the authors are trying to solve.*
Response to R2.2 We added a simulation experiment that demonstrates the sensitivity of our model in comparison to the other two methods. Simulation methods, results and a figure were added to the manuscript. Through this experiment, we have shown that for total counts, regularized negative binomial outperforms the other two models in sensitivity and specificity. For unique counts, the regularized negative binomial model has higher sensitivity but slightly lower specificity when the condition mean becomes closer to the control mean. In general, sensitivity and accuracy drop as the condition mean counts becomes closer to the control mean counts for all models as expected.

Comment R2.3 *Lines 100-8: I find this formulation (used through the manuscript), to be fairly confusing. G is the set of genes, but also the set of genetic backgrounds (which is true for a specific type of background, single-gene knockouts). Doesn't R depend on g & e?*
Response to R2.3 We agree with the reviewer that the formulation was a bit confusing. We now formulated genes under investigation as $\mathcal{S}$ instead of $\mathcal{G}$ to avoid confusion with the genetic background $g \in \mathcal{G}$. We defined $\mathcal{R}$ as a set of beneficial and detrimental genes such that the false discovery rate is bounded. This set might vary depending on the genetic background (g) or environmental condition (e) under investigation.

Comment R2.4 *Finally, the constant reference to "essential/dispensable" is confusing. It would seem from the preceding text that it should read "essential" only (although, later we see that deviations can be in both directions, which is what I think motivates this presentation).*
Response to R2.4 This comment parallels Reviewer 1 comment R1.2. We have streamlined the narrative of the paper and rigorously defined the mathematical sets that the terms are based upon as described in that comment.

Comment R2.5 *Line 59: probably a better phrasing would be "robust to differences in transposon sequencing technology".*
Response to R2.5 We modified the phrasing according to the reviewers suggestion.

Comment R2.6 *Fig 4: Can the authors add the raw number of genes (not just percentages) to the diagrams?*
Response to R2.6 We added the raw number of genes besides the percentages to the Fig 4.

Comment R2.7 *Lines 370-80: Can the authors add the overlap to their gene counts? For example, when they present "337/395" for the heat shock case, how many genes are in the overlap between total and unique?*
Response to R2.7 We added the counts of the overlapping genes for the Tn-seq experiment as per the reviewers suggestion.

References

[1] M. A. DeJesus, S. Nambi, C. M. Smith, R. E. Baker, C. M. Sassetti, and T. R. Ioerger. Statistical analysis of genetic interactions in Tn-Seq data. *Nucleic Acids Res*, 45(11):e93, Jun 2017.
