# Supplementary material for: Model-based identification of conditionally-essential genes from transposon-insertion sequencing data
Source: PLoS Comput Biol. 2022 Mar 7;18(3):e1009273. doi: 10.1371/journal.pcbi.1009273 (PMC8929702; doi:10.1371/journal.pcbi.1009273)
Supplement: Attachment — Submitted filename: ploscomp_reviewers_letter__minor.pdf [file pcbi.1009273.s008.pdf]

Answers to Reviewer 1

Comment R1.1 *The authors have responded thoughtfully to my previous comments, and I have no further comments or concerns.*
Response to R1.1 We thank the reviewer for providing valuable feedback which improved our manuscript.

Answers to Reviewer 2

Comment R2.1 *The newly added "Sensitivity, Specificity, and Accuracy" indeed does address the comment I raised in the previous round, but it is very hard to follow. The description should be rewritten to be clearer or a cartoon representation of the simulated settings could be added for clarity.*
Response to R2.1 We thank the reviewer for their comment. We now have rewritten our text on how we simulated data for the sensitivity experiments. For clarity, we also added a Supplementary Figure 2 for the diagrammatical representation of simulated data.

Comment R2.2 *The supplementary figures are misnumbered. I think both the flowchart (referred to as SFig 1 in the text) and the results of the sensitivity experiments (referred to as SFig 3) could be moved to the main text.*
Response to R2.2 We have corrected our supplementary figure numbering. We have also moved the hierarchical decision tree representation of gene categories to the main text (Fig 1). Sensitivity, Specificity, and Accuracy results figure is now added to the main text (Fig 4).

Comment R2.3 *Generally speaking, the figures would benefit from some minor polish for readability: the fonts are too small, sometimes abbreviations are used when there appears to be enough room to spell out the terms, some of the alignments seem off (particularly in supplementary figure 3).*
Response to R2.3 We have improved the readability of text and alignments in all of the figures notably the figure of simulation results (now Fig 4) as the reviewer pointed out.
